# Comparison of Four Intrapartum Cardiotocography Classifications for Predicting Neonatal Acidemia at Birth

**DOI:** 10.1155/2023/5853889

**Published:** 2023-02-13

**Authors:** Nika Troha, Katja Razem, Ursa Luzovec, Miha Lucovnik

**Affiliations:** ^1^Department of Perinatology, Division of Obstetrics and Gynecology, University Medical Center Ljubljana, Ljubljana, Slovenia; ^2^Department of Obstetrics and Gynecology, General Hospital Slovenj Gradec, Slovenj Gradec, Slovenia; ^3^Medical Faculty, University of Ljubljana, Ljubljana, Slovenia

## Abstract

**Objective:**

To compare diagnostic values of four intrapartum cardiotocography (CTG) classifications in predicting neonatal acidemia at birth.

**Methods:**

Retrospective case-control study. Forty-three CTG traces with an umbilical artery pH < 7.00 (study group) and 43 traces with a pH ≥ 7.00 (control group) were analyzed. Inclusion criteria were singleton pregnancy, cephalic presentation, admission to labour ward during active phase of first stage of labour, and gestational age 37^+0^ to 41^+6^ weeks. Exclusion criteria were suspected intrauterine growth restriction, oligohydramnios, polyhydramnios, pregestational or gestational insulin-dependent diabetes mellitus, and preeclampsia. Last 30-60 minutes before delivery of CTG traces was classified retrospectively according to four classification systems—International Federation of Gynecology and Obstetrics (FIGO), Royal College of Obstetricians and Gynaecologists (RCOG), National Institute of Child Health and Human Development (NICHD), and the 5-tier system by Parer and Ikeda. Predictive value of each classification for neonatal acidemia was assessed using receiver operating characteristics (ROC) analysis.

**Results:**

FIGO, RCOG, and NICHD classifications predicted neonatal acidemia with areas under the ROC curves (AUC) of 0.73, 95% confidence interval (CI) 0.63-0.84; 0.72, 95% CI 0.60-0.83; and 0.69, 95% CI 0.57-0.80, respectively. The five-tier system by Parer and Ikeda had significantly better predictive value with an AUC of 0.96, 95% CI 0.91-1.00.

**Conclusions:**

The 5-tier classification system proposed by Parer and Ikeda for assessing CTG in labour was superior to FIGO, RCOG, and NICHD intrapartum CTG classifications in predicting severe neonatal acidemia at birth.

## 1. Introduction

Cardiotocography (CTG) has become an established method of fetal monitoring since its introduction in the late 1950s [1]. It currently represents the gold standard for fetal surveillance during labour despite lack of firm evidence that its use prevents neonatal mortality and long-term morbidity caused by hypoxic-ischemic injury [1, 2]. High inter- and intraobserver variability in interpretation of CTG traces is one of the main limitations of this intrapartum fetal monitoring methodology [3–5]. To address this shortcoming, several CTG classification systems have been developed during the last decades with an intent to make CTG interpretation more objective and consistent [6].

Currently, classifications proposed by the International Federation of Gynecology and Obstetrics (FIGO), Royal College of Obstetricians and Gynaecologists (RCOG), National Institute of Child Health and Human Development (NICHD), and the 5-tier system by Parer and Ikeda are most widely used. Tables 1 and 2 present CTG parameter definitions and classification criteria in these four classification systems. Only few studies to date, however, compared predictive values of various classifications. Coletta et al. found the 5-tier system to better predict umbilical artery pH < 7.00 compared with the NICHD 3-tier system but have not studied predictive values of other classifications [7]. More recently, Di Tommaso et al. found relatively poor predictive values of all CTG classifications studied, including Parer and Ikeda's 5-tier, the NICHD's 3-tier, and RCOG's classifications, for neonatal pH < 7.15 [8]. They have not, however, included fetuses/neonates with severe acidosis (pH < 7.00), which is truly associated with neonatal mortality and morbidity such as hypoxic-ischemic encephalopathy [9].

The objective of our study was to compare predictive values of four commonly used intrapartum CTG classifications for predicting severe neonatal acidemia at birth.

## 2. Methods

This was a single-center, retrospective, case-control study. CTG tracings from patients delivering at our tertiary perinatal center between 2016 and 2020 with neonatal umbilical artery pH < 7.00 at birth were matched to patients without fetal/neonatal academia (umbilical artery pH at birth > 7.00) by the following parameters: singleton pregnancies, with cephalic fetal presentation, at term (between 37^+0^ and 41^+6^ weeks of pregnancy), admission to labour ward during active phase of first stage of labour (regular contractions every 5 minutes with evidence of cervical ripening) without suspected intrauterine growth restriction, oligohydramnios (amniotic fluid index (AFI) < 5 cm or single deepest vertical (SDP) pocket of amniotic fluid < 2 cm), polyhydramnios (AFI > 25 cm and SDP > 8 cm), and gestational or preexisting insulin-dependent diabetes mellitus and preeclampsia. All births fulfilling inclusion criteria with neonatal pH < 7.00 during the study period were included in the study group. As more births were candidates for controls, we included consecutive births fulfilling matching criteria with neonatal pH > 7.00 as controls until the same number of births was reached in both groups (1 matched control per case).

At our institution, CTG is routinely monitored throughout labour and umbilical blood collected at birth for acid-base analysis in all deliveries. Blood gases are measured in a calibrated automated analyzer (ABL80 Flex, Radiometer, Denmark) with a pH difference of 0.02 for estimation of arterial and venous umbilical blood samples. Last 30-60 minutes before delivery of selected CTG tracings was retrospectively reviewed and classified according to four intrapartum CTG classifications (FIGO, RCOG, NICHD, and 5-tier classification by Parer and Ikeda) by three obstetricians (K.R., U.L., and M.L.) certified in CTG interpretation. The time limit of 30-60 min before delivery was used due to its best reflection of neonatal pH and was adjusted according to the specific classification system's definitions [7]. Internal CTG transducer was used in case of inadequate quality of external monitoring in the absence of its contraindications. Traces were classified using definitions and terminology of each classification. Reviewers were blinded to maternal and neonatal clinical characteristics including umbilical artery pH results at birth. Each reviewer classified CTG tracings independently. In cases of discordant classification, they reviewed, discussed, and classified the tracing together reaching consensus. When classification process was completed, electronic medical records were used to obtain maternal and fetal clinical information.

For continuous variables, data were expressed as median with 25th and 75th percentiles. Categorical data were summarized as frequencies and percentages. For comparison between the two study groups (neonatal umbilical artery pH < 7.00 vs. ≥ 7.00), Mann–Whitney *U* test was used for continuous variables and Chi-square test or Fisher's exact test for categorical variables, as appropriate. Receiver operating characteristic (ROC) curves were used to evaluate capacity of each classification to predict neonatal acidemia. Areas under the ROC curve (AUC) were calculated with 95% confidence intervals (CI). Predictive value of a classification was considered low with an AUC < 0.7, moderate with an AUC 0.7–0.9, and high with an AUC > 0.9. Sensitivity, specificity, positive likelihood ratio, negative likelihood ratio, positive predictive value, and negative predictive value for neonatal acidemia were evaluated for each category within each classification system. The Fleiss kappa coefficient (*κ*) was calculated to assess the interobserver reliability in classifying CTG parameters for each classification system. Statistical analysis was performed using IBM SPSS Statistics for Windows version 28.0.1.1 (14) (Armonk, NY: IBM Corp.).

The National Medical Ethics Committee approved the study (reference number: 0120-65/2017-3; KME 60/03/17).

## 3. Results

We identified 43 CTG tracings from patients with an umbilical artery pH < 7.00 and 43 controls (pH ≥ 7.00) fulfilling our inclusion criteria.  Table 3 presents baseline maternal characteristics and modes of delivery in the two groups. External CTG transducer was used in two cases (5%) in the umbilical artery pH < 7.00 at birth group and none in the pH > 7 group. In 18 cases, the indication for operative delivery (cesarean section or operative vaginal delivery) was suspected fetal acidemia based on CTG pattern. Neonatal umbilical artery pH was <7.00 in 11 (61%) and ≥ 7.00 in 7 (39%) of these cases.

Table 4 presents neonatal characteristics and outcomes. Besides lower umbilical artery pH, the Apgar scores at 1 and 5 minutes were significantly lower in neonates with umbilical artery pH < 7.00.

In 66 (77%) CTG traces, all three reviewers were unanimous in their classification without discussing the trace. In 20 (23%) cases, consensus was reached after discussion. Overall, the interobserver agreement for classifying a CTG tracing was substantial for the FIGO (*κ* = 0.76), RCOG (*κ* = 0.71), and Parer and Ikeda (*κ* = 0.68) classifications and almost perfect (*κ* = 0.82) for the NICHD classification. Type of decelerations, by which the trace could be classified in different categories, was the object of discussion in all cases. Proportions of CTG tracings in each classification category in the acidemia vs. no-acidemia groups are presented in Table 5.

**Figure 1:**
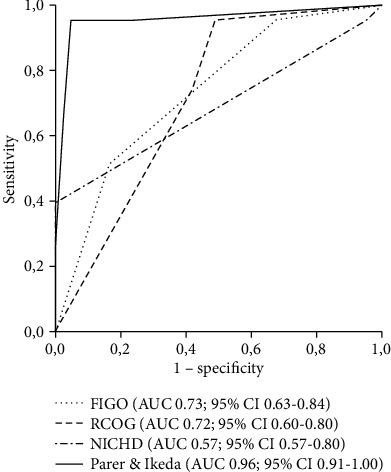
Comparison of receiver operating characteristics (ROC) curves for four different intrapartum cardiotocography (CTG) classifications to predict neonatal acidemia (umbilical artery pH < 7) at birth. AUC: area under the ROC curve; CI: confidence interval.

Analysis of ROC curves showed low to moderate discriminative capacity in prediction of fetal/neonatal acidemia for FIGO (AUC 0.73; 95% CI 0.63-0.84), RCOG (AUC 0.72; 95% CI 0.60-0.80), and NICHD (AUC 0.69; 95% CI 0.57-0.80) classifications. Parer and Ikeda classification had an excellent predictive value with an AUC of 0.96 (95% CI 0.91-1.00) (Figure 1).

Table 6 presents diagnostic values (sensitivity, specificity, positive/negative likelihood ratios, and positive/negative predictive values) for each category within the four classifications. Among all categories in the four classifications analyzed, the green and blue categories in the Parer and Ikeda classification had the highest sensitivities (95.4% and 100%, respectively). Yellow, orange, and red Parer and Ikeda categories also had the highest specificities among all specific categories (97.6%, 97.7%, and 100%, respectively). Similarly, positive and negative likelihood ratios in all Parer and Ikeda categories as well as positive and negative predictive values were higher compared to other classifications.

## 4. Discussion

Among four of currently most widely used CTG classifications, the 5-tier classification by Parer and Ikeda seems to have the best predictive value for identifying fetuses with severe acidemia during labour. While we found only low to moderate discriminative capacity in prediction of severe neonatal acidemia at birth for FIGO, RCOG, and NICHD classifications, the 5-tier classification predicted neonatal umbilical artery pH < 7.00 very accurately with an area under the ROC curve of 0.96.

Our results are in accordance with several studies published to date. When comparing the 5-tier proposed by Parer and Ikeda and the 3-tier NICHD classification, Coletta et al. found that the 5-tier system performed better in identifying fetuses at risk for acidemia [7]. These results were recently confirmed by Kikuchi et al. [10]. Gyamfi Bannerman et al. also found the 5-tier classification to be superior to the 3-tier NICHD classification with a 79% sensitivity and 100% specificity for an umbilical artery pH of <7.00 [11]. In their study, 8.3% of tracings with severe acidemia were classified as “green” [11]. This is similar to the 5% of tracings classified as “green” and also to 5% of tracings classified as normal according to other classifications in fetuses with pH < 7.00 in our study. Usefulness of the 5-tier classification is further corroborated by Japanese experience with countrywide extensive adoption of this method for classifying and managing CTG tracings in labour [12]. Katsuragi et al. found a sevenfold decrease in incidence of metabolic acidemia without a concurrent increase operative delivery rates following introduction of the 5-tier classification [13, 14]. In addition, Elliot et al. demonstrated that the degree and duration of CTG abnormality, defined by the 5-tier system and analyzed using a specialized software, correlated with fetal metabolic acidemia and neurologic injury [15].

While there is plenty of data demonstrating superiority of the 5-tier classification over the 3-tier classification proposed by the NICHD, only few studies compared the 5-tier classification to other CTG classification systems mostly used outside the United States today. In 2013, Di Tommaso et al. compared predictive values of five CTG classifications for diagnosis of fetal/neonatal academia (defined in their study as umbilical artery pH < 7.15) [8]. Besides the 5-tier and the 3-tier classification, they also analyzed CTG classifications proposed by RCOG, Dublin Fetal Heart Rate Monitoring Trial (DFHRMT), and the Society of Obstetricians and Gynaecologists of Canada (SOGC) [8]. Similarly to our study, they found Parer and Ikeda's 5-tier classification to have the best “trade-off” between sensitivity and specificity. However, in contrast to our results, they found low or moderate predictive values for low neonatal umbilical artery pH in all classifications. Contrarily to our study, however, they only evaluated a sample of fetuses with milder acidemia (pH > 7.0 and < 7.15). This could explain the much lower predictive value of the 5-tier classification observed in their study compared to our results. A case-control study published in 2016 compared the FIGO CTG classification system with the 5-tier system by Parer and Ikeda [16]. Gamboa et al. found both classifications to have comparable diagnostic accuracy for mild and severe acidemia. This is in contrast with our results, which indicate a significantly better predictive value of the 5-tier system compared to the FIGO classification for diagnosing severe neonatal academia.

Only few studies to date compared predictive values of other frequently used CTG classifications. Santo et al. evaluated accuracy of CTG interpretation in prediction of newborn acidemia using the FIGO, *American College of Obstetricians and Gynecologists* (ACOG), and *National Institute for Health and Care Excellence* (NICE) guidelines interpreted by 27 observers. FIGO and NICE guidelines achieved higher sensitivities compared to ACOG (89 and 97% vs. 32%) whereas ACOG guidelines showed a significantly higher specificity (95%) than FIGO (63%) or NICE (66%) guidelines. According to the authors, the reason for the higher specificity of ACOG classification is due to the fact that ACOG has more restrictive criteria for classifying tracings to category III (to the pathological category) compared to the other two CTG guidelines. Some of the cases of acidemia classify that category II consequently the sensitivity of the guideline is lower and specificity is higher than in the other classifications [17]. Even more recently, Zamora del Pozo et al. compared FIGO, ACOG, NICE, and Chandraharan guidelines' predictive value for neonatal acidemia with three independent observers. Chandraharan guidelines had the highest discrimination capacity for neonatal acidemia (AUC 0.66; 95% CI, 0.55-0.77), but it did not differ significantly from the other guidelines. As with the former study, ACOG reached the highest specificity (95.73%) among the classifications, while Chandraharan guidelines reached the highest sensitivity (78.79%) [18].

Strengths of our study include the blinded review of CTG tracings, universal cord gas collection, and an index group defined by umbilical artery pH < 7.0 where the median base excess was -18.1. This is a clinically meaningful index group with umbilical artery gas values well below the 2nd percentile that are associated with significantly elevated risks of newborn encephalopathy, identifying features associated with true pathology. Our study also has several limitations. We only included 43 CTG traces with neonatal pH < 7.00. This reflects the low incidence of severe fetal academia among term uncomplicated pregnancies. Our results could, therefore, be due to small number of cases included and should be confirmed or refuted by further studies. We chose to include three different CTG reviewers given the well describer interobserver variability in CTG interpretation. In this way, we managed to avoid studying predictive values of CTG classifications based solely on one clinician's ability to interpret and classify CTG tracings. However, in everyday clinical practice, we do not always have the luxury of three different reviewers discussing ambiguous cases and reaching consensus. Clinical applicability of our results should, therefore, be tested in prospective studies. This is especially true since the higher predictive value of the 5-tier classification could be due to the fact that traces can be classified in more than just three categories. This could make interpretation and classification more cumbersome and could even increase inter- and intraobserver variability. Our results support this, since further discussion among CTG reviewers was necessary in 23% of cases to reach consensus on type of decelerations. Interobserver agreement for classifying CTG traces was the lowest for the 5-tier Parer and Ikeda classification. Due to its complexity, the 5-tier system could, therefore, require specific staff training and adjustment. Further research is also needed to assess whether subcategorizing the second category of the 3-tier system might produce similar results.

Many decades have been needed for experts and different professional societies to agree on basic definitions of normal and abnormal CTG parameters. The current challenge is to translate this consensus into uniform classification of CTG tracings, which could eventually lead to a more standardized intrapartum CTG management. Different classification systems are now being used in different parts of the world. Our study was designed to compare the performance of four methods of grading intrapartum CTG tracings using visual inspection and existing rule-based methods that are in clinical use. This research question is important because CTG classification systems are often adopted/promoted without data describing their predictive values, let alone justifying one over another. This kind of comparisons is essential for clinicians given the potential for devastating sequelae of delayed intervention and subsequent fetal brain injury based on false reassurance from a certain classification of the tracing or conversely the potential for excessive unnecessary interventions based on an overly sensitive classification method. Our study indicates that among the most widely used CTG classifications today, the 5-tier classification proposed by Parer and Ikeda has the highest discriminative capacity in prediction of neonatal acidemia.

## Figures and Tables

**Table 1:** Comparison of the basic fetal heart rate (FHR) definitions contained in the International Federation of Gynecology and Obstetrics (FIGO) guidelines of 2015, Royal College of Obstetricians and Gynaecologists (RCOG) of 2001, National Institute of Child Health and Human Development (NICHD) of 2008, and Parer and Ikeda's 5-tier classification system of 2007.

Cardiotocography (CTG) feature		FIGO	NICHD	RCOG	Parer and Ikeda's 5-tier system
Baseline	Definition	Mean level of FHR when this is stable, accelerations and decelerations being absent. It is determined over a time period of 5 or 10 min and expressed in bpm	Baseline FHR is the approximate mean FHR rounded to increments of 5 bpm during a 10-minute segment	Mean level of the FHR when this stable, excluding accelerations and decelerations. It is determined over a time period of 5 or 10 min and expressed in bpm	Mean FHR, rounded off to 5 bpm in 10 min tracing segment with exclusion of periodic and episodic FHR changes and increased variability
	Normal	110-160 bpm	110-160 bpm	110–160 bpm	110-160 bpm
	Tachycardia	>160 bpm	>160 bpm	>180 bpm (161–180 bpm is moderate tachycardia)	>160 bpm
	Bradycardia	<110 bpm	< 110 bpm	<100 bpm (100–109 bpm is moderate bradycardia)	<110 bpm

Variability	Definition	Oscillations in the FHR signal, evaluated as the average bandwidth amplitude of the signal in 1-minute segments	Fluctuations in the baseline FHR of 2 cycles per minute or greater	Minor fluctuations in baseline FHR occurring at three to five cycles per minute. It is measured by estimating the difference in bpm between the highest peak and lowest point of fluctuation in a 1 min segment of the trace	Oscillation in baseline FHR, uneven in amplitude and frequency. Visually assessed as an amplitude range from the highest to the lowest 1 min FHR frequency
	Normal	5−25 bpm	6-25 bpm	5-25 bpm	6-25 bpm
	Reduced	< 5 bpm for more than 50 minutes in baseline segments or > 3 minutes during decelerations	*<*5 bpm	<5 bpm for 40–90 min (nonreassuring) or > 90 min (abnormal variability)	<5 bpm
	Increased	A bandwidth value exceeding 25 bpm lasting more than 30 minutes	>25 bpm	Nonreassuring: >25 bpm lasting 15-25 min Abnormal: >25 bpm lasting >25 min	>25 bpm

Accelerations	Definition	Abrupt (from onset of acceleration to peak in less than 30 seconds) increases in FHR above the baseline, of more than 15 bpm in amplitude and lasting more than 15 seconds but less than 10 minutes	Visually apparent abrupt increase (from onset of acceleration to peak in less than 30 seconds) in FHR above the baseline	Transient increases in FHR of ≥15 bpm and lasting ≥15 sec	Visually apparent and rapid increase in FHR > 15 bpm above baseline FHR lasting 15 sec (up to 2 min). Prolonged acceleration: 2-10 min

Decelerations	Definition	Decreases in the FHR below the baseline of more than 15 bpm in amplitude and lasting more than 15 seconds	—	Transient episodes of slowing of FHR below the baseline level of >15 bpm and lasting ≥15 sec or more	—
	Early	Shallow, short-lasting decelerations, with normal variability within the deceleration, that are coincident with contractions	Visually apparent gradual decrease (defined as onset of deceleration to nadir ≥30 seconds) and return to baseline FHR associated with a uterine contraction	Uniform, repetitive, periodic slowing of FHR with onset early in the contraction and return to baseline at the end of the contraction	Visually apparent and gradual (≥30 sec from onset to nadir) symmetrical decline in FHR and return to baseline FHR. Onset, nadir, and return to baseline FHR coincide with onset, peak, and end of contraction
	Variable	Decelerations that exhibit a rapid drop (onset to nadir in less than 30 seconds), good variability within the deceleration, rapid recovery to the baseline, varying in size, shape, and relationship to uterine contractions	Visually apparent abrupt (< 30 sec from the onset of the deceleration to the beginning of the FHR nadir) decrease in FHR	Variable, intermittent periodic slowing of FHR with rapid onset and recovery. Time relationships with contraction cycle are variable, and they may occur in isolation. Sometimes they resemble other types of deceleration patterns in timing and shape	An abrupt (< 30 sec from onset to nadir) decrease in FHR of ≥15 bpm below baseline lasting ≥15 sec (up to 2 min). Their onset, depth, and duration commonly vary with successive uterine contractions *Moderate*: <70 bpm lasting 30-60 sec or < 80 bpm lasting >60 sec *Severe*: < 70 bpm lasting >60 sec or < 80 bpm lasting >60 sec Other decelerations are considered as *mild*.
	Late	U-shaped and/or with reduced variability are decelerations with a gradual onset and/or a gradual return to the baseline and/or reduced variability within the deceleration	Visually apparent usually symmetrical gradual (from the onset to the FHR nadir of >30 seconds) decrease and return of FHR associated with a uterine contraction	Uniform, repetitive periodic slowing of FHR with onset mid to end of the contraction and nadir >20 s after the peak of the contraction and ending after the contraction. In the presence of a nonaccelerative trace with baseline variability < 5 bpm, the definition would include decelerations < 15 bpm	Visually apparent, gradual decrease (>30 s after onset to nadir) and return to baseline FHR. The nadir of the deceleration occurring after the peak of the contraction *Mild*: FHR decline up to 15 bpm below baseline *Moderate*: FHR decline from 16 to 44 bpm below baseline *Severe*: FHR decline ≥45 bpm below baseline
	Prolonged	Deceleration lasting >3 minutes	Visually apparent decrease in FHR below the baseline. The decrease from the baseline is >15 bpm, lasting >2 minutes, but <10 minutes from onset to return to baseline	An abrupt decrease in FHR to levels below the baseline that lasts at least 60–90 s. These decelerations become pathological if they cross two contractions (i.e., >3 min)	Abrupt decrease in FHR below baseline lasting ≥2-10 min. *Moderate*: <70 bpm; *severe*: 70-80 bpm

Sinusoidal pattern	Definition	A regular, smooth, undulating signal, resembling a sine wave, with an amplitude of 5−15 bpm and a frequency of 3−5 cycles per minute. This pattern lasts more than 30 minutes and coincides with absent accelerations.	Sine wave-like pattern of regular frequency and amplitude and is excluded in the definition of FHR variability	A regular oscillation of the baseline long-term variability resembling a sine wave. This smooth, undulating pattern, lasting at least 10 min, has a relatively fixed period of three to five cycles per minute and an amplitude of 5–15 bpm above and below the baseline. Baseline variability is absent	Visually apparent, smooth, sine wave-like undulating pattern in FHR baseline with a cycle frequency of 3–5/min that persists for >20 minutes

FHR: fetal heart rate; bpm: beats per minute.

**Table 2:** Cardiotocography classification criteria in the International Federation of Gynecology and Obstetrics (FIGO) guidelines of 2015, Royal College of Obstetricians and Gynaecologists (RCOG) of 2001, National Institute of Child Health and Human Development (NICHD) of 2008, and Parer and Ikeda's 5-tier classification system of 2007 guidelines.

Classification	Interpretation	Baseline fetal heart rate	Variability	Decelerations and accelerations
FIGO	Normal	110-160 bpm	5-25 bpm	No repetitive (if they occur at >50% contractions) decelerations
	Suspicious	Lacking at least one of normal characteristics but with no pathological features		
	Pathological	<100 bpm	Reduced increased variability, sinusoidal pattern	Repetitive late or prolonged decelerations lasting >30 min (or 20 min if reduced variability) or one prolonged deceleration lasting >5 min

NICHD	Category I (includes all the following)	110-160 bpm	6-25 bpm	Late or variable decelerations absent Early decelerations present or absent Accelerations present or absent
	Category II	Includes all tracings not categorized as category I or III		
	Category III	Absent baseline variability and any of the following: recurrent late decelerations or recurrent variable decelerations Bradycardia (FHR < 110 bpm) Sinusoidal pattern		

RCOG	Normal (a CTG where all of the following four reassuring features are present)	110-160 bpm	5-25 bpm	No decelerations Accelerations present.
	Suspicious (a CTG with features categorized under one of the nonreassuring categories and the remainder of the features categorized under the reassuring category)	100–109 bpm 161–180 bpm	<5 bpm exceeding 40 min and less than 90 min	Typical variable decelerations with over 50% of contractions occurring for over 90 minutes Single prolonged deceleration for up to 3 minutes The absence of accelerations with an otherwise normal trace is of uncertain significance
	Pathological (a CTG with one or more of the following features or two or more features in the previous category)	<100 bpm >180 bpm Sinusoidal pattern for more than 10 minutes	<5 bpm exceeding 90 min	Late decelerations A commonly found variable decelerations Single prolonged deceleration exceeding 3 min

Parer and Ikeda's 5-tier classification	Green	110-160 bpm	6-25 bpm	Early or mild VD
	Blue	Moderate variability	Minimal variability (<5 bpm)	Absent variability/other baseline characteristics
		Tachycardia with early or mild VD Normal baseline with mild or moderate VD	Tachycardia without decelerations Normal baseline +/- early decelerations	—
	Yellow	Tachycardia with moderate VD, mild/moderate LD or PD Normal baseline with severe VD, moderate/severe LD, mild/moderate PD Mild bradycardia +/- early, mild/moderate VD, LD, or PD Moderate bradycardia +/- early decelerations	Tachycardia with early or mild VD Normal baseline with mild VD	Marked variability (>25 bpm)
	Orange	Tachycardia with severe VD, LD, or PD Normal baseline with severe PD Mild bradycardia with severe VD, LD, or PD Moderate bradycardia with severe VD, LD, or PD Any severe bradycardia	Tachycardia with moderate/severe VD, moderate/mid VD or PD Normal baseline with moderate/severe VD, moderate/mid VD or PD Mild or moderate bradycardia +/- early deceleration	Normal baseline
	Red	—	Tachycardia with severe LD Normal baseline with severe LD or PD Mild or moderate or severe bradycardia with VD, LD, and PD	Any baseline with any deceleration Sinusoidal pattern

FHR: fetal heart rate; LD: late decelerations; VD: variable decelerations; PD: prolonged decelerations.

**Table 3:** Maternal characteristics and mode of delivery.

Characteristic/mode of delivery	pH < 7.00 (*N* = 43)	pH ≥ 7.00 (*N* = 43)	*p* value
Maternal age (years)	30 (26, 35)	31 (28, 35)	0.805
Nulliparity	23 (53%)	20 (47%)	0.51
Maternal prepregnancy BMI (kg/m^2^)	24 (22, 27)	23,5 (21, 27)	0.24
Maternal BMI at delivery (kg/m^2^)	29 (27, 32)	28 (26, 31)	0.339
Cesarean delivery	8 (19%)	6 (14%)	0.559
Operative vaginal delivery	3 (7%)	1 (2%)	0.61

BMI: body mass index. Data are presented as median with 25th and 75th percentiles or *n* (%).

**Table 4:** Neonatal characteristics and outcomes.

Characteristic/outcome	pH < 7.00 (*N* = 43)	pH ≥ 7.00 (*N* = 43)	*p* value
Birth weight (g)	3500 (3200, 3770)	3540 (3290, 3960)	0.358
Umbilical artery pH	6.99 (6.94, 7.00)	7.28 (7.21, 7.32)	<0.001^∗^
Umbilical vein pH	7.06 (7.00, 7.13)	7.35 (7.29, 7.39)	<0.001^∗^
Umbilical artery BE	-18.1 (-19.6, -16.8)	-5.1 (-9.2, -3.6)	<0.001^∗^
Apgar 1 min	8 (7, 9)	9 (9, 9)	<0.001^∗^
Apgar 5 min	9 (8, 9)	9 (9, 9)	<0.001^∗^
Apgar (1 min) 0-3	2 (5%)	1 (2%)	1.0
Apgar (1 min) 4-6	6 (14%)	1 (2%)	0.11
Apgar (1 min) 7-10	35 (81%)	41 (95%)	0.089
Apgar (5 min) 0-3	0	0	—
Apgar (5 min) 4-6	3 (7%)	1 (2%)	0.61
Apgar (5 min) 7-10	40 (93%)	42 (98%)	0.616
NICU admission	7 (16%)	2 (5%)	0.156
Need for resuscitation	8 (19%)	1 (2%)	0.03^∗^
Need for assisted ventilation	1 (2%)	0	1.0
Cooling therapy	1 (2%)	0	1.0
Neonatal encephalopathy presentation	1 (2%)	0	1.0

BE: base excess; NICU: neonatal intensive care unit. Data are presented as median with 25th and 75th percentiles or *n* (%); ^∗^ represents statistical significance.

**Table 5:** Classification of cardiotocographic (CTG) traces according to neonatal umbilical artery pH at birth.

	pH < 7.00	pH ≥ 7.00
FIGO		
Normal	2 (5%)	14 (32%)
Suspicious	19 (44%)	22 (51%)
Pathological	22 (51%)	7 (16%)
RCOG		
Normal	2 (5%)	22 (51%)
Suspicious	9 (21%)	3 (7%)
Pathological	32 (74%)	18 (42%)
NICHD		
Category I (normal)	2 (5%)	2 (5%)
Category II (intermediate)	24 (56%)	41 (95%)
Category III (abnormal)	17 (39.5%)	0
Parer and Ikeda		
Green	2 (5%)	33 (76%)
Blue	0	8 (19%)
Yellow	13 (30%)	1 (2%)
Orange	17 (39.5%)	1 (2%)
Red	11 (25.5%)	0

FIGO: International Federation of Gynecology and Obstetrics; RCOG: Royal College of Obstetricians and Gynaecologists; NICHD: National Institute of Child Health and Human Development.

**Table 6:** Diagnostic values of categories within classifications for neonatal acidemia.

	Sensitivity	Specificity	Positive likelihood ratio	Negative likelihood ratio	Positive predictive value	Negative predictive value
FIGO						
Normal	95.3%	32.6%	1.41	0.14	58.6%	87.5%
Suspicious	44.2%	48.8%	0.86	1.14	46.3%	46.5%
Pathological	51.2%	83.7%	3.14	0.58	75.9%	67.4%
RCOG						
Normal	95.3%	51.2%	1.95	0.09	66.1%	91.7%
Suspicious	20.9%	93%	3	0.85	75%	54%
Pathological	74.4%	58.1%	1.78	0.44	64%	69.4%
NICHD						
Category I (normal)	95.4%	4.7%	1	1	50%	50%
Category II (intermediate)	55.8%	95.3%	12	0.46	92.3%	68.3%
Category III (abnormal)	39.5%	100%	—	0.6	100%	62.3%
Parer and Ikeda						
Green	95.4%	76.7%	4.1	0.06	80.4%	94.3%
Blue	100%	81.4%	5.4	0	84.3%	100%
Yellow	30.2%	97.6%	13.0	0.7	92.9%	58.3%
Orange	39.5%	97.7%	17.0	0.6	94.4%	61.7%
Red	25.6%	100%	—	0.4	100%	57.3%

FIGO: International Federation of Gynecology and Obstetrics; RCOG: Royal College of Obstetricians and Gynaecologists; NICHD: National Institute of Child Health and Human Development.

## Data Availability

The data used to support the findings of this study are available from the corresponding author upon request.
